# An update on malignant tumor-related stiff person syndrome spectrum disorders: clinical mechanism, treatment, and outcomes

**DOI:** 10.3389/fneur.2023.1209302

**Published:** 2023-10-04

**Authors:** Yong Peng, Huan Yang, Ya-hui Xue, Quan Chen, Hong Jin, Shu Liu, Shun-yu Yao, Miao-qiao Du

**Affiliations:** ^1^Department of Neurology, Affiliated First Hospital of Hunan Traditional Chinese Medical College, Zhuzhou, Hunan, China; ^2^The Third Affiliated Hospital of Hunan University of Chinese Medicine, Zhuzhou, Hunan, China; ^3^Department of Neurology, Xiangya Hospital, Central South University, Changsha, Hunan, China

**Keywords:** stiff person syndrome (SPS), stiff person syndrome spectrum disorders (SPSSDs), paraneoplastic, cancer, malignant, autoantigen

## Abstract

Stiff person syndrome (SPS) is a rare central nervous system disorder associated with malignancies. In this review, we retrieved information from PubMed, up until August 2023, using various search terms and their combinations, including SPS, stiff person syndrome spectrum disorders (SPSSDs), paraneoplastic, cancer, and malignant tumor. Data from peer-reviewed journals printed in English were organized to explain the possible relationships between different carcinomas and SPSSD subtypes, as well as related autoantigens. From literature searching, it was revealed that breast cancer was the most prevalent carcinoma linked to SPSSDs, followed by lung cancer and lymphoma. Furthermore, classic SPS was the most common SPSSD subtype, followed by stiff limb syndrome and progressive encephalomyelitis with rigidity and myoclonus. GAD65 was the most common autoantigen in patients with cancer and SPSSDs, followed by amphiphysin and GlyR. Patients with cancer subtypes might have multiple SPSSD subtypes, and conversely, patients with SPSSD subtypes might have multiple carcinoma subtypes. The first aim of this review was to highlight the complex nature of the relationships among cancers, autoantigens, and SPSSDs as new information in this field continues to be generated globally. The adoption of an open-minded approach to updating information on new cancer subtypes, autoantigens, and SPSSDs is recommended to renew our database. The second aim of this review was to discuss SPS animal models, which will help us to understand the mechanisms underlying the pathogenesis of SPS. In future, elucidating the relationship among cancers, autoantigens, and SPSSDs is critical for the early prediction of cancer and discovery of new therapeutic modalities.

## 1. Introduction

Stiff person syndrome (SPS) is a rare chronic central nervous system (CNS) disorder (1). The clinical manifestations of SPS encompass a wide range of symptoms, including muscle rigidity, sporadic muscle spasms, and chronic muscle pain. It is also characterized by psychiatric symptoms, such as depression and anxiety, and also other neurological symptoms, including horizontal and vertical supranuclear gaze palsy, nystagmus, increased reflexes, and paroxysmal dysautonomic crisis (2, 3).

Recently, SPS spectrum disorders (SPSSDs) have expanded to include a series of diseases with similar signs and symptoms to those of SPS (4).

SPS is associated with malignancies; however, this is not really well-understood. In this review, we retrieved information from PubMed, up until August 2023, using various search terms and their combinations, including SPS, SPSSDs, paraneoplastic, cancer, and malignant. Data from peer-reviewed journals printed in English were organized to explain the possible relationships between different carcinomas and SPSSD subtypes, as well as related autoantigens. An analysis of the literature search revealed that breast cancer was the most prevalent carcinoma linked to SPSSDs, followed by lung cancer and lymphoma. The first aim of this review highlights the complex nature of the relationships among cancers, autoantigens, and SPSSDs as new information in this field continues to be generated globally. The adoption of an open-minded approach to updating information on new cancer subtypes, autoantigens, and SPSSDs is recommended to renew our database. The second aim of this review was to outline SPS animal models, which will help us to understand the mechanisms of pathogenesis of SPS. In future, elucidating the relationship among cancers, autoantigens, and SPSSDs is critical for the early prediction of cancer and the discovery of new therapeutic modalities.

## 2. Major clinical characteristics of SPSSDs

SPS was first reported by Moersch and Woltman in 1956 (1). In 1999, Brown et al. published the “Diagnostic Criteria for Classic Stiff-Person Syndrome,” which classified SPS into the following two major subtypes: (1) classic SPS, cases without encephalomyelitis; and (2) SPS plus, cases with encephalomyelitis, such as progressive encephalomyelitis with rigidity and myoclonus (PERM), jerking stiff man syndrome, and stiff limb syndrome (SLS) (5). Currently, SPS includes the following three subtypes: (1) glutamic acid decarboxylase 65 (GAD65)-positive SPS associated with other autoimmune conditions; (2) anti-amphiphysin-positive SPS associated with tumors; and (3) seronegative idiopathic SPS (6).

To date, SPSSDs include the following: (1) partial SPS, limited to extremities and often only one limb (stiff limb syndrome, SLS) or the torso; (2) SPS-plus, with classic SPS symptoms that exist in combination with cerebellar and/or brainstem findings; (3) PERM; and (4) some overlapping syndromes, such as classic SPS with epilepsy or limbic encephalitis (LE) (7), classic SPS with myasthenia gravis (8), classic SPS with anti-N-methyl-D-aspartate receptor (NMDAR) encephalitis (NMDARE-SPS) (9), classic SPS with central sleep apnea (10), and classic SPS with pure red blood cell aplasia (11). Most patients with SPSSD are middle-aged females; however, some patients with SPSSD are either pediatric individuals or adult males. For example, among a total of 22 patients, eight older male patients with SPSSD showed early prominent vestibular and ocular motor dysfunction (4, 12).

Several autoantigens are associated with SPSSD. The major SPSSD autoantibodies are antibodies against GAD, amphiphysin, and glycine receptors for PERM (13). GAD65 is the major autoantibody associated with SPSSD and is linked to classic SPS (4, 14). Other autoantigens, such as glycine receptors (linked to PE RM) (15, 16), amphiphysin (linked to cancers) (17), GABAA receptors (18) and its related protein GABAA receptor-associated protein (GABARAP) (19), dipeptidyl-peptidase-like protein-6 (DPPX), and Zic4 (linked to small-cell lung cancer) (20) are also associated with SPSSD. In addition, SPSSD is associated with breast cancer, small-cell lung cancer, and lymphoma (4, 21). Recently, SPSSD has also been reported to be associated with some rare cancers (21–36). In this review, we summarize the current literature on malignant tumor-related SPSSDs.

## 3. Clinical characteristics of malignant tumor-related SPSSDs

### 3.1. Breast cancer

Breast cancer is the most common carcinoma linked to SPSSDs. Table 1 shows that from 29 studies on breast cancer, six SPSSD subtypes, including classic SPS (21, 30, 37–47, 50, 63, 64), SLS (57, 58, 65), paraneoplastic cerebellar degeneration (59), subacute sensory neuronopathy, subacute cerebellar degeneration (60), and PERM (61), among which classic SPS is the major SPSSD subtype, were found to be involved. Patients with breast cancer and PSSD were determined to have other carcinomas, such as colon cancer, non-Hodgkin lymphoma, thymoma and lymphoma, and malignant melanoma (21, 34, 39, 41). Furthermore, patients with breast cancer and SPSSD were found to have other diseases, including autoimmune diseases, such as paraneoplastic encephalomyelitis, type 1 diabetes, thyroid disease, pernicious anemia, vertigo, psoriasis, thyroid disease, rheumatoid arthritis, sarcoidosis, mixed connective disease, limbic encephalitis, myelopathy, HIV, and ischemic cardiomyopathy (21, 34, 41, 45, 58, 59, 65). Amphiphysin (55) is the most common autoantigen in patients with breast cancer and SPSSD, followed by GAD65, Ri, acetylcholine receptor (AChR), and glycine receptor (GlyR). Notably, Connolly et al. reported a 53-year-old male patient with breast cancer and classic SPS who harbored the GAD65 autoantibody (42).

**Table 1:** Breast cancer associated with SPSSD.

**SPSD subtype**	**References**	**Gender**	**Number**	**Age**	**Countries or regions**	**Autoantibodies**	**Coexisting with other diseases**
Classic SPS	Wessig et al. (37)	Female	1	71	Germany	Amphiphysin	Unknown
	Schmierer et al. (38)	Female	1	53	UK	Amphiphysin	Unknown
	Nene et al. (39)	Female	1	58	USA	GAD65	Colon cancer
	Thümen et al. (40)	Female	1	58	Germany	Ri	Unknown
	Lemieux et al. (41)	Female	1	30	Canada	Unknown	Paraneoplastic encephalomyelitis
	McKeon et al. (21)	Unknown	6	Unknown	USA	GAD65	Non-Hodgkin lymphoma, Type 1 diabetes, Thyroid disease, Pernicious anemia Vitiligo, Other antibody detected
	Connolly et al. (42)	Male	1	34	USA	GAD65	Unknown
	Rojas-Marcos et al. (43)	Unknown	1	Unknown	Spain	Amphiphysin	Unknown
	Dogruoz Karatekin et al. (44)	Female	1	45	Turkey	Amphiphysin	Unknown
	Ibrikji et al. (45)	Female	1	49	Lebanon	Amphiphysin	Transverse myelitis, hypothalamitis
	Huang et al. (46)	Female	1	56	China	GAD65	Unknown
	Piccolo et al. (30)	Unknown	3	54–60	Italy	AchR	Unknown
	Vinjam et al. (47)	Female	1	47	UK	Amphiphysin, GAD65	Unknown
	Kelly et al. (48)	Female	1	64	USA	Amphiphysin	Unknown
	Carvajal-González (34)	Unknown	2	Unknown	UK, Germany, Sweden, Belgium	GlyR	Thymoma and lymphoma, Hodgkin lymphoma, malignant melanoma, thymoma, B cell marginal zone lymphoma associated with monoclonal gammapathy igm, metastases from previous treated breast cancer, psoriasis, thyroid disease, diabetes, rheumatoid arthritis; sarcoid; mixed connective disease
	Vacaras et al. (28)	Female	1	68	Romania	Amphiphysin	Unknown
	Folli et al. (49)	Female	3	54-76	Italy, UK	Amphiphysin	Unknown
	Rosin et al. (50)	Female	1	59	Germany	Amphiphysin, GAD65	Unknown
	Sinnreich et al. (51)	Female	1	85	Switzerland	GAD65	Unknown
	Petzold et al. (52)	Female	1	62	Germany	Amphiphysin	Rhabdomyolysis
	Kocak (53)	Female	1	71	USA	Amphiphysin	Unknown
	Pittock et al. (54)	Female	15	46-80	USA, Korea, Sweden	Amphiphysin	Unknown
	De Camilli et al. (55)	Unknown	4	Unknown	USA, Germany, UK	Amphiphysin	Unknown
	Floyd et al. (56)	Female	1	44	USA	Amphiphysin	Unknown
SLS	Agarwal et al. (57)	Female	1	55	India	GAD65	Unknown
	Krishna et al. (58)	Female	1	54	USA	Amphiphysin	Limbic encephalitis
PCD	Khanam et al. (59)	Female	1	67	USA	VGCC	HIV, ischemic cardiomyopathy
SSN	Aydin et al. (60)	Female	2	36 - 40	Turkey	Hu, Zic4	Unknown
SCD	Aydin et al. (60)	Female	1	53	Turkey	Yo	Unknown
PERM	De Blauwe et al. (61)	Female	1	66	Belgium	Glycine Receptor	Unknown
	Antoine et al. (62)	Female	1	75	France, USA	Amphiphysin	Unknown

### 3.2. Lung cancer

Lung cancer has also been linked to SPSSD. This has been reported in nine studies, involving six SPSSD subtypes, including classic SPS (66–69), subacute sensory neuronopathy, subacute cerebellar degeneration (60), paraneoplastic neurologic syndromes (70), and PERM (71–73) (Table 2). Sinha et al. reported that thymoma coexists with lung cancer and SPSSD (76). Table 2 shows that GAD65 is the most common autoantigen reported in patients with lung cancer and SPSSD, followed by amphiphysin, GABABR, and Hu.

**Table 2:** Lung cancer associated with SPSSD.

**SPSD subtype**	**References**	**Gender**	**Number**	**Age**	**Countries or regions**	**Autoantibodies**	**Coexisting with other diseases**
Classic SPS	Dropcho et al. (66)	M:F 1:2	3	52–67	Germany	Amphiphysin	Unknown
	Boronat et al. (67)	Unknown	29	Unknown	Spain	GABA_B_R	Unknown
	Sarwari et al. (68)	Female	1	41	USA	GAD65	Unknown
	Lester et al. (69)	Female	1	64	Mexico	AChRGN, GAD65	Unknown
SSN	Aydin et al. (60)	Female	1	42	Turkey	Hu	Unknown
SCD	Aydin et al. (60)	Male	1	69	Turkey	Hu	Unknown
CIPO	Badari et al. (70)	Male	1	61	USA	Hu	Unknown
PERM	Kyskan et al. (71)	Male	1	39	Canada	Gly-R	Unknown
	Nguyen-Huu et al. (72)	Female	1	75	Germany	Amphiphysin	Unknown
	Spitz et al. (73)	Male	1	73	Brasil	GAD65	Unknown
LEMS	Abboud et al. (74)	Female	1	68	USA	PQ-VGCC	Unknown
	Ray and Nigam (75)	Female	1	75	UK	Unknown	Unknown

### 3.3. Lymphoma and similar hematological carcinomas

Lymphoma and similar hematological carcinomas have been reported to be associated with SPSSD. In total, 10 studies involving three SPSSD subtypes, such as classic SPS (21, 34, 77–80), SLS (81), and PERM (82–84), have been reported (Table 3). Some authors have reported the coexistence of thymoma and breast cancer with lymphoma and SPSSD (34, 80). Table 3 shows that GlyR is the most commonly reported autoantigen in patients with lymphoma and similar hematological carcinomas and SPSSD, followed by GAD65, PCA-1, PCA-Tr, and striational antibodies.

**Table 3:** Lymphoma and similar hematological carcinomas associated with SPSSD.

**SPSD subtype**	**References**	**Gender**	**Number**	**Age**	**Countries or regions**	**Autoantibodies**	**Coexisting with other diseases**
Classic SPS	Rakocevic et al. (77)	Female	1	57	USA	GAD65	Unknown
	Nuti et al. (78)	Male	1	70	Italy	GlyR	Unknown
	McKeon et al. (85)	Unknown	1	Unknown	USA	GlyR	Unknown
	Gutmann et al. (79)	Female	1	52	Italy	Unknown	Unknown
	Tsai et al. (80)	Male	1	66	Australia	GAD65	Thymoma
	Carvajal-Gonzalez et al. (34)	Unknown	3	Unknown	UK, Germany, Sweden, Belgium	GlyR	Thymoma, breast cancer, psoriasis, thyroid disease, diabetes, rheumatoid arthritis; sarcoid; mixed connective disease
SLS	Derksen et al. (81)	Male	1	61	Germany	GlyR	Unknown
PERM	Borellini1 et al. (82)	Male	1	60	Italy	GlyR	Unknown
	Schmidt et al. (83)	Female	1	21	Germany	Unknown	Unknown
	Tchapyjnikov et al. (84)	Male	1	18	USA	Unknown	Unknown

### 3.4. Other carcinomas

SPSSD is also associated with other carcinomas, such as mediastinal liposarcoma (22), metastatic adenocarcinoma (23), pancreatic adenocarcinoma (24), renal cell carcinoma (25), mediastinal cancer, undifferentiated carcinoma of an undetermined origin (26), multiple myeloma (86), embryonal carcinoma (27), malignant glioma (87), ovarian adenocarcinoma (88), prostate carcinoma (88), testicular seminoma and germ cell neoplasia (88), pancreatic cancer (88), melanoma (88), invasive carcinoma of no special type (28), ovarian teratoma (9), small cell carcinoma of the bladder (31), pleuropulmonary blastoma (33), malignant mesothelioma (35), colon cancer, and Hürthle cell adenoma (36). It is also associated with overlapping cancers, such as breast cancer with colon cancer (30, 39), chronic lymphocytic leukemia (81), thymoma and non-Hodgkin lymphoma (80), non-functioning pituitary microadenoma, and endometrial cancer (29) (Table 4). Table 5 shows the other carcinomas included in 25 studies involving six SPSSD subtypes, namely, classic SPS (21–36), SLS (86, 90), PERM (91), progressive dizziness and unstable gait (87), and NMDAR-SPS (9). Furthermore, thyroid and renal cell cancers reportedly coexist with colon cancer and SPSSD (21). Table 4 shows that GAD65 is the most common autoantigen in patients with other carcinomas and SPSSD, followed by anti-nuclear, Ri, NCC-ST 439, amphiphysin, gephyrin, AchR, anti-islet cell, VGKC-complex, and LGI1 antigens.

**Table 4:** Other carcinomas associated with SPSSD.

**SPSD subtype**	**References**	**Carcinoma**	**Gender**	**Number**	**Age**	**Countries or regions**	**Autoantibodies**	**Coexisting with other diseases**
Classic SPS	Yohannan et al. (22)	Mediastinal liposarcoma	Female	1	20s	USA	GAD65	Seizure
	McCabe et al. (23)	Metastatic adenocarcinoma	Female	1	43	UK	Anti-nuclear, Ri	Unknown
	Yong et al. (28)	Pancreatic Adenocarcinoma	Female	1	70	Singapore	GAD65	Unknown
	McHugh et al. (25)	Renal cell carcinoma	Male	1	53	Ireland	GAD65	Unknown
	Butler et al. (26)	Mediastinic canceran undifferentiated carcinoma	Male	1	58	USA	Anti-nuclear, NCC-ST 439, Gephyrin	Unknown
	McKeon et al. (22)	Thyroid, renal cell, and colon cancer	Unknown	59	Unknown	USA	GAD65	Unknown
	Komandla et al. (27)	Embryonal carcinoma	Male	1	34	USA	Amphiphysin	Unknown
	Vacaras et al. (28)	An invasive no special type carcinoma	Female	1	68	Romania	Amphiphysin	Unknown
	Yeoh et al. (29)	Non-functioning pituitary microadenoma, endometrial cancer	Female	1	53	USA	ANA, GAD65	Unknown
	Piccolo et al. (30)	Colon cancer	Unknown	3	54 - 60	Italy	AchR	Unknown
	Alboniga-Chindurza et al. (31)	Small cell carcinoma of the bladder	Male	1	46	Spain	GAD65	Unknown
	Hylan et al. (32)	Colon cancer	Female	1	56	USA	Unknown	Unknown
	Jun et al. (33)	Pleuropulmonary Blastoma	Female	1	**3.5**	Korea	Unknown	Unknown
	Carvajal-Gonzalez et al. (34)	Malignant melanoma (21, 34, 39, 41)						Unknown
	Koca et al. (35)	Malignant mesothelioma	Female	1	58	Turkey	GAD65	Unknown
	Badzek et al. (36)	Colon cancer, Hürthle cell adenoma	Female	1	55	Croatia	GAD65	Unknown
	Clow et al. (89)	Multiple myeloma	Female	1	31	Canada	GAD65	Unknown
SLS	Silverman et al. (90)	Metastatic adenocarcinoma						Unknown
	Schiff et al. (86)	Multiple myeloma	Female	1	47	USA	GAD65, anti-islet cell	Unknown
PERM	Shugaiv et al. (91)	Renal cell carcinoma	Male	1	46	Spain	GAD65, VGKC-complex, LGI1	Unknown
Progressive dizziness and unstable gait	Maimaiti et al. (87)	Malignant glioma	Male	1	62	China	GAD65	Unknown
NMDAR-SPS	Gharedaghi et al. (9)	Ovarian teratoma	Female	1	26	USA	NMDAR, GAD65	Unknown

**Table 5:** GAD65 associated with cancer or SPSSD.

**SPSD subtype**	**Carcinoma**	**References**	**Coexist with other cancer**	**Coexist with other autoantigen**
Classic SPS	Breast cancer	(21, 39, 42, 46, 47, 50, 51, 56)	Colon cancer, non-Hodgkin lymphoma	Amphiphysin
	Thymoma	(11, 80, 92–99)	Non-Hodgkin lymphoma	Amphiphysin
	Lung cancer	(68, 69, 73)	Unknown	Unknown
	Lymphoma	(77, 80)	Thymoma	Unknown
	Mediastinal liposarcoma	(22)	Unknown	Unknown
	Metastatic adenocarcinoma	(23)	Unknown	Unknown
	Pancreatic adenocarcinoma	(24)	Unknown	Unknown
	Renal cell carcinoma	(100)	Unknown	Unknown
	Thyroid, renal cell, and colon cancer	(21)	Unknown	Unknown
	Non-functioning pituitary microadenoma, endometrial cancer	(29)	Unknown	ANA
	Small cell carcinoma of the bladder	(31)	Unknown	Unknown
	Malignant melanoma	(34)	Unknown	Unknown
	Malignant mesothelioma	(35)	Unknown	Unknown
	Colon cancer, Hürthle cell adenoma	(36)	Unknown	Unknown
	Multiple myeloma	(89)	Unknown	Unknown
	Metastatic adenocarcinoma	(90)	Unknown	Unknown
	Multiple myeloma	(86)	Unknown	Anti-islet cell
MG-SPS	Thymoma	(8, 55, 101–103)	Unknown	AchR, gastric parietal cell, ssDNA dsDNA
SLS	Breast cancer	(57)	Unknown	Unknown
PERM	Thymoma	(104–106)	Unknown	AchR
	Renal cell carcinoma	(91)	Unknown	VGKC-complex, LGI1
Progressive dizziness and unstable gait	Malignant glioma	(87)	Unknown	Unknown
NMDAR-SPS	Ovarian teratoma	(9)	Unknown	NMDAR

## 4. Possible mechanisms of paraneoplastic SPSSD

As we believe that autoantigens might be good candidates for determining the possible mechanism underlying paraneoplastic SPSSD, we have summarized the detailed information on autoantigens, including GAD and amphiphysin, followed by GlyR, gephyrin, anti-islet cell, and LGI1 (please see Supplementary Table 1).

### 4.1. GAD

#### 4.1.1. GAD isoform

GAD is predominantly expressed in neurons, which might be linked to SPSSD, and insulin-secreting pancreatic β cells, which might be linked to type I diabetes (107). GAD regulates the decarboxylation of glutamate to gamma-aminobutyric acid (GABA), the main inhibitory neurotransmitter within the CNS, and is related to SPSSD (108, 109). There are two GAD isoforms—GAD65 and GAD67. GAD65 is expressed in the presynaptic end of nerve terminals in its inactive form and is converted to its active form at the post-natal stage to rapidly synthesize GABA for synaptic transmission (110). GAD65 is also responsible for packaging GABA after its synthesis (111). Additionally, GAD67 is expressed in the cell body and dendrites and is responsible for synthesizing basal levels of GABA (100).

GAD65 antibody (Ab) titers and epitope specificities are present in different diseases and different subtypes of SPSSD (112, 113). For example, the GAD65 Ab titer is 348 U/mL in type I diabetes, 6.0 × 105 U/mL in cerebellar ataxia (CA), 6.2 × 105 U/mL in LE, and 1.1 × 106 U/mL in SPS (112). GAD65 binding in the presence of rFab b78 is 99% in type I diabetes, 81% in CA, 88% in LE, and 77% in SPS (112). Moreover, positive GAD immunoreactivity is ≥1,800 U/mL in SPS rat brain sections as detected *via* immunohistochemistry or cell-based assays (7, 114). A high range of GAD65 Ab levels is associated with SPS, whereas a lower one is associated with type I diabetes (13). A possible mechanism underlying this distinction could be that the GAD Ab in type I diabetes primarily reacts with conformational epitopes, whereas GAD antibodies in SPS recognize linear epitopes (115–117). Furthermore, GAD Ab-positive type I diabetes or SPS, CA, and LE are associated with different HLA class II haplotypes (118–121).

#### 4.1.2. Decreased GABAergic activity is the major physiopathological mechanism of SPSSD

GABAergic neurons are responsible for inhibitory signals in the CNS and express high levels of GAD65. They are mainly located in the hippocampus, cerebellum, basal ganglia, brainstem nuclei, and spinal gray matter (122, 123). GABA binds GABAA and GABAB receptors to mediate the hyperpolarization of post-synaptic neurons, comprising an inhibitory signal (124, 125). The GAD Ab inhibits GAD65 to block GABA synthesis, thereby reducing the uptake of newly synthesized GABA in synaptic vesicles and its synaptic release (111, 126–128).

Inhibiting GABA synthesis results in decreased GABAergic transmission, which is linked to neuronal hyperexcitability and is the core pathophysiological mechanism in SPS (129, 130). For example, the possible mechanism underlying SPS might be mediated by the inhibition of GABAergic neurons in the spinal cord, resulting in a state of motor neuron hyperexcitability, ultimately causing the simultaneous contraction of agonist and antagonist muscles (100, 131). GABAergic interneurons are located at different levels of the CNS, other than the spinal cord, leading to other subtypes of SPSSD, such as PERM (128), which has also been supported by animal studies (132, 133). However, this is not the case for LE and temporal lobe epilepsy, owing to insufficient data.

#### 4.1.3. Association with carcinomas or SPSSD Subtypes

The major SPSSD subtype is classic SPS (11, 68, 69, 73, 77, 80, 92–98, 134, 135), followed by SPS with myasthenia gravis (21–25, 29, 31, 34–36, 86, 89, 90, 135, 136), PERM (91), SLS (57), progressive dizziness and unstable gait (87), and NMDAR-SPS (9), as shown in Table 5. Moreover, the major carcinoma associated with SPSSD subtypes is breast cancer (21, 39, 42, 47, 50, 51, 56, 57, 137), followed by thymoma (11, 80, 91–98, 104–106, 134, 135), lymphoma (77, 80), lung cancer (68, 69, 73), and other carcinomas (21–25, 29, 31, 34–36, 86, 89, 90, 135, 136).

#### 4.1.4. Titer of anti-GAD65 Ab in the serum vs. the cerebrospinal fluid of patients with SPSSD

One report showed that the median concentration of anti-GAD65 Ab, measured *via* ELISA, is 30-fold higher in the serum (74,700 IU/mL) than in the cerebrospinal fluid (CSF) (2,430 IU/mL). However, these data were from 34 patients with classical anti-GAD65-associated syndromes, including SPS, CA, chronic epilepsy, and LE, with overlapping syndromes in some of the cases (138). The serum/CSF ratio of anti-GAD65 Ab was reported to be approximately 20 in patients with SPS (138). Moreover, serum and CSF anti-GAD65 Ab titers decreased, with those of CSF decreasing more rapidly than serum titers after patients with SPS received immunotherapy (138).

### 4.2. Amphiphysin

#### 4.2.1. Amphiphysin superfamily

Amphiphysins are members of the Bin-Amphiphysin-Rvsp (BAR) family of proteins, which includes the mammalian bridging-integrators (Bin1 and Bin2), amphiphysins, and yeast Rvs161p and Rvs167p (139). Some members of the amphiphysin superfamily have conserved BAR domains, mainly in the N-terminus, and an SH3 domain in the C-terminus (139). Amphiphysin I is expressed in chicken and mammalian brains (140) and is associated with SPS and breast cancer (49, 55). Two members of amphiphysin II are also expressed in the brain. Amphiphysin II, also known as BIN1 (MYC box-dependent interacting protein-1 or bridging integrator-1) or SH3P9, is associated with cancer progression, several myopathies, heart failure, and late-onset Alzheimer's disease (141). Amphiphysin IIa shares a brain-specific domain with amphiphysin I (142, 143), and amphiphysin IIb has a skeletal muscle-specific domain with a tumor suppressor that interacts with the c-Myc oncoprotein (142, 144). In several cancers, such as breast, colon, prostate, and lung cancers, as well as hepatocarcinoma and neuroblastoma, the expression of amphiphysin II is reduced or altered (145–148). In addition, the ablation of amphiphysin II is linked to a poor cancer prognosis and increased metastasis (145, 148–151). Amphiphysin II can also inhibit Myc-dependent transformation and tumorigenesis (145, 148–151).

#### 4.2.2. Association with carcinomas or SPSSD subtypes

The major SPSSD subtype is classic SPS (28, 37, 38, 43–45, 47–50, 52–56, 135), followed by SLS (58, 65) and PERM (62, 72). Moreover, the major carcinoma associated with SPSSD is breast cancer (28, 37, 38, 43–45, 47–50, 52–56, 58, 135), followed by thymoma (66, 95, 99) and lung cancer (66, 72) (Table 6).

**Table 6:** Amphiphysin associated with cancer or SPSSD.

**SPSD subtype**	**Carcinoma**	**References**	**Coexist with other cancer**	**Coexist with other autoantigen**
Classic SPS	Breast cancer	(28, 37, 38, 43–45, 47, 49, 50, 52–55, 96, 140)	Unknown	GAD65
	Lung cancer	(66)	Unknown	Unknown
SLS	Breast cancer	(58, 65)	Unknown	Unknown
PERM	Breast cancer	(62)	Unknown	Unknown
	Lung cancer	(72)	Unknown	Unknown

### 4.3. Glycine receptors

#### 4.3.1. Biological studies on GlyR

As an inhibitory neurotransmitter, glycine, as well as its receptor (GlyR), is critical for CNS development (152). Glycine is synthesized via serine hydroxymethyl transferase or a glycine synthase (glycine cleavage, GCS) enzyme, located between carbon dioxide, ammonium ion, N5, N10-methylene tetrahydrofolate, NADH, and a proton, producing glycine, tetrahydrofolate, and NAD+ (153), as confirmed from a rat study (154). Furthermore, the biological function of glycine requires specific transporters such as GlyT1 (glial cells) and GlyT2 (neurons) (155, 156). GlyT1 also regulates glutamatergic neurotransmission through NMDA receptors, affecting brain function and diseases (157).

There are four α subunits and one β unit in GlyR, and these are expressed in the spinal cord and retina, respectively (158–160). Microglia secrete glycine, enhance NMDA receptor-mediated responses (161), and express GlyR to induce membrane depolarization, increasing intracellular calcium and proliferation (162). In addition, glial cells modulate synaptic development by participating in the induction of the action potential conduction in white matter via GlyRs (163). Importantly, glycine has also been linked to rapid cancer cell proliferation due to glycine metabolism (164). For example, α1 and α3 GlyR subunits were found to be expressed in human brain tumor biopsies, and the lack of α1 GlyR protein expression resulted in inhibition of the self-renewal capacity and tumorigenicity of GL261 glioma cells (165). GlyR knockdown can increase P53 tumor suppressor protein expression (166, 167).

### 4.4. Association with carcinomas or SPSSD Subtypes

The major associated SPSSD subtype has been reported to be classic SPS (34, 78, 85), followed by SLS (61) and PERM (61, 82) (Table 7). Moreover, the major associated carcinoma is lymphoma (34, 61, 78, 85), followed by breast cancer (34, 61).

**Table 7:** GlyR associated with cancer or SPSSD.

**SPSD subtype**	**Carcinoma**	**References**	**Coexist with other cancer**	**Coexist with other autoantigen**
Classic SPS	Breast cancer	(34)	Unknown	GAD65
	Lymphoma	(21, 34, 78)	Unknown	Unknown
SLS	Lymphoma	(81)	Unknown	Unknown
PERM	Breast cancer	(61)	Unknown	Unknown
	Lymphoma	(82)	Unknown	Unknown

## 5. Clinical characteristics of malignant tumor-related SPSSDs

### 5.1. Breast Cancer

Breast cancer is the most common carcinoma linked to SPSSDs. Table 1 shows that from 29 studies on breast cancer, six SPSSD subtypes, including classic SPS (21, 30, 37–47, 50, 63, 64), SLS (57, 58, 65), paraneoplastic cerebellar degeneration (59), subacute sensory neuronopathy, subacute cerebellar degeneration (60), and PERM (61), among which classic SPS is the major SPSSD subtype, were found to be involved. Patients with breast cancer and PSSD were determined to have other carcinomas, such as colon cancer, non-Hodgkin lymphoma, thymoma and lymphoma, and malignant melanoma (21, 34, 39, 41). Furthermore, patients with breast cancer and SPSSD were found to have other diseases, including autoimmune diseases, such as paraneoplastic encephalomyelitis, type 1 diabetes, thyroid disease, pernicious anemia, vertigo, psoriasis, thyroid disease, rheumatoid arthritis, sarcoidosis, mixed connective disease, limbic encephalitis, myelopathy, HIV, and ischemic cardiomyopathy (21, 34, 41, 45, 58, 59, 65). Amphiphysin (55) is the most common autoantigen in patients with breast cancer and SPSSD, followed by GAD65, Ri, acetylcholine receptor (AChR), and glycine receptor (GlyR). Notably, Connolly et al. reported a 53-year-old male patient with breast cancer and classic SPS who harbored the GAD65 autoantibody (42).

### 5.2. Lung cancer

Lung cancer has also been linked to SPSSD. This has been reported in nine studies, involving six SPSSD subtypes, including classic SPS (66–69), subacute sensory neuronopathy, subacute cerebellar degeneration (60), paraneoplastic neurologic syndromes (70), and PERM (71–73) (Table 2). Sinha et al. reported that thymoma coexists with lung cancer and SPSSD (76). Table 2 shows that GAD65 is the most common autoantigen reported in patients with lung cancer and SPSSD, followed by amphiphysin, GABABR, and Hu.

### 5.3. Lymphoma and similar hematological carcinomas

Lymphoma and similar hematological carcinomas have been reported to be associated with SPSSD. In total, 10 studies involving three SPSSD subtypes, such as classic SPS (21, 34, 77–80), SLS (81), and PERM (82–84), have been reported (Table 3). Some authors have reported the coexistence of thymoma and breast cancer with lymphoma and SPSSD (34, 80). Table 3 shows that GlyR is the most commonly reported autoantigen in patients with lymphoma and similar hematological carcinomas and SPSSD, followed by GAD65, PCA-1, PCA-Tr, and striational antibodies.

### 5.4. Other carcinomas

SPSSD is also associated with other carcinomas, such as mediastinal liposarcoma (22), metastatic adenocarcinoma (23), pancreatic adenocarcinoma (24), renal cell carcinoma (25), mediastinal cancer, undifferentiated carcinoma of an undetermined origin (26), multiple myeloma (86), embryonal carcinoma (27), malignant glioma (87), ovarian adenocarcinoma (88), prostate carcinoma (88), testicular seminoma and germ cell neoplasia (88), pancreatic cancer (88), melanoma (88), an invasive carcinoma of no special type (28), ovarian teratoma (9), small cell carcinoma of the bladder (31), pleuropulmonary blastoma (33), malignant mesothelioma (35), colon cancer, and Hürthle cell adenoma (36). It is also associated with overlapping cancers, such as breast cancer with colon cancer (30, 39), chronic lymphocytic leukemia (81), thymoma and non-Hodgkin lymphoma (80), non-functioning pituitary microadenoma, and endometrial cancer (29) (Table 4). Table 5 shows the other carcinomas included in 25 studies involving six SPSSD subtypes, namely, classic SPS (21–36), SLS (86, 90), PERM (91), progressive dizziness and unstable gait (87), and NMDAR-SPS (9). Furthermore, thyroid and renal cell cancers reportedly coexist with colon cancer and SPSSD (21). Table 4 shows that GAD65 is the most common autoantigen in patients with other carcinomas and SPSSD, followed by anti-nuclear, Ri, NCC-ST 439, amphiphysin, gephyrin, AchR, anti-islet cell, VGKC-complex, and LGI1 antigens.

## 6. Treatment and outcomes of paraneoplastic SPSSD

For patients with paraneoplastic SPSSD, the carcinoma is typically detected and identified prior to treatment while concurrently managing and addressing symptoms.

### 6.1. GABAergic therapy

In patients with SPSSD, antibodies attack the GAD enzyme, which is essential for GABA production. Therefore, drugs targeting GABAergic neurons can be effective in treating SPSSD; by inhibiting the attack on GAD, GABA levels are reduced (168).

#### 6.1.1. Benzodiazepines

Benzodiazepines are the first-line treatment for patients with SPS. These drugs enhance the neurotransmitter effect of GABA at its receptor. Furthermore, benzodiazepines are widely used for their sedative, muscle-relaxant, and anticonvulsant effects (21). Long-term benzodiazepine therapy has been shown to benefit patients with classic or partial SPS and reduce GAD-65-positive Ab-mediated stiffness and spasm symptoms; however, this improvement might also be due to other adjunct medications.

The major drug for SPSSD treatment is diazepam, which results in a good response in most patients at high doses of up to 60 mg daily (169). However, owing to concerns about withdrawal from long-term use and high doses of diazepam therapy, tizanidine has emerged as a good candidate for alternative therapy. As an NMDAR, tizanidine is an α2 inhibitor that inhibits glutamate release and prevents glutamatergic hyperactivity, thereby resolving convulsions in patients with SMS. Nevertheless, the dose of tizanidine should be individualized (21, 169).

#### 6.1.2. Baclofen

Baclofen is an agonist of GABA type B receptors that inhibits reflexive muscle contraction by blocking the release of excitatory neurotransmitters through voltage-gated calcium channels (170). It is also a second-line therapy for patients with SPS. However, to date, the use of oral baclofen therapies is still being debated. In one report, high doses (however, the dose is unknown) of oral baclofen therapy were found to result in serious side effects, such as sedation and respiratory depression (171). However, oral baclofen had good effects on SPS patients without serious side effects. For example, oral baclofen therapy (5 mg, three times per day) plus clonazepam resulted in improvements in a 69-year-old man with SPS and amphiphysin antibodies (172). Symptomatic treatment initiated with oral clonazepam and baclofen (5 mg Bid), followed by intravenous immunoglobulin (IVIG) resulted in improvements in a 60-year-old man with SPS associated with critical illness polyneuropathy (173). Baclofen (30 mg/day) combined with oral diazepam and steroids resulted in improvements in a 55-year-old GAD-Ab-positive female patient with SLS and breast carcinoma (57). For childhood-onset SMS, three SMS patients had good clinical responses with oral baclofen (dose range, 60–80 mg) combined with diazepam, IVIG, plasma exchange or dantrolene, and botulinum toxin (174).

Alternatively, intrathecal therapy is an effective route for baclofen treatment (2). The chronic infusion of intrathecal baclofen can improve SPS patient outcomes, including the pain Numeric Rating Scale, Spasm Frequency Scale, and lower extremity Modified Ashworth Scale (171). Intrathecal baclofen (100 μg) followed by a rehabilitation program resulted in substantial clinical and functional improvements in a 59-year-old female SPS patient, who had no therapeutic response with oral benzodiazepines and botulinum toxin injections (175). In addition, intrathecal baclofen (started from 50 μg/d up to 100 μg/d) improved motor functions in a 48-year-old male GAD-negative SPS patient (176). Baclofen can be used to effectively treat SPS because it is a direct agonist of GABA-B receptors and does not require endogenous GABA to induce presynaptic inhibition (176).

#### 6.1.3. Levetiracetam

Levetiracetam binds to synaptic vesicle glycoprotein 2A (SV2A), resulting in the release of the neurotransmitter stored within the vesicle, rapidly inhibiting firing neurons and potassium and N-type calcium channels (177, 178). In a previous study, three patients with high anti-GAD65 Ab levels did not respond satisfactorily to IVIG and diazepam treatment with or without plasmapheresis (179). These patients were treated with 500 mg oral levetiracetam twice daily, which improved axial rigidity and the disappearance of paroxysmal respiratory arrest within 3 days of therapy initiation, with markedly reduced leg stiffness and ameliorated walking difficulties (179). However, to date, there is no evidence of the effects of long-term levetiracetam therapy. The possible mechanism by which levetiracetam achieves its effects could be by stabilizing and strengthening GABAA and decreasing hyperexcitability in spinal cord neurons (179).

#### 6.1.3. Pregabalin

Structurally, pregabalin is classified as a GABA analog or gabapentinoid (180). In a previous study, a female patient with SMS who did not respond to diazepam treatment, owing to excessive sedation, was successfully treated with a 3-month pregabalin regimen (181). The possible mechanism underlying the effects of pregabalin might be the inhibition of calcium influx and subsequent release of excitatory neurotransmitters, including glutamate and norepinephrine, resulting in compensation for the imbalance between inhibitory and excitatory intracortical circuits (181).

#### 6.1.4. Propofol

The mechanism of action of propofol in the CNS is unclear. Propofol might enhance the function of GABA receptors, evoking the chloride current in central neurons at clinically relevant concentrations, ultimately activating the GABA receptor–chloride ionophore complex (182). Notably, a low dose of propofol improves symptoms in patients with SPS who do not respond to high-dose benzodiazepines, baclofen, corticosteroids, levetiracetam, IVIG, or IV ethanol. Furthermore, propofol is effective for patients with SMS that is refractory to therapy (183). Unfortunately, long-term propofol therapy has unsatisfactory effects in patients with SPS (184).

### 6.2. Immunotherapy

#### 6.2.1. Rituximab

Rituximab binds to the CD20 antigen on mature B cells, leading to B cell lysis, while sparing precursor B cells. Rituximab improves SPS and other neurological autoimmune disorders, such as Devic's disease, myasthenia gravis, autoimmune neuropathies, and inflammatory myopathies (185). SPS is associated with elevated titers of anti-GAD65 Abs and glycine receptor α-subunits in patients (186). Four reports have demonstrated the benefits of rituximab for patients with SPS (186–189). Although rituximab improved the clinical conditions of patients, the decrease in the anti-GAD titer was inconsistent in different reports. Some reports demonstrated that after rituximab treatment, the anti-GAD titer was rapidly (17 days, from positive to undetectable) or slowly (1 year, from 1,000 to 400 U/mL) reduced (187). However, another case report showed that the anti-GAD Ab titer remained elevated, even during treatment with rituximab (188).

#### 6.2.2. Tacrolimus

Tacrolimus inhibits the calcium calcineurin pathway and exerts its immunosuppressive effect by reducing the proliferation of activated T cells (190). Furthermore, tacrolimus decreases IL-2 levels and impairs T-helper cell functions, finally reducing the activation of B cells to produce antibodies. It also suppresses the function of anti-GAD Abs, thereby blocking GABAergic neurotransmission and interfering with GABA synthesis (191). Tacrolimus directly blocks calcineurin in the GABAergic inhibitory system. Nonetheless, the neuroprotective effect of tacrolimus therapy on SPS demonstrated based on the reduced density of neurons with somal areas and improved pathological conditions, remains debatable (192); evidence that macrolide antibiotics inhibit the function of immunophilins and provide neuroprotective and neuroregenerative effects contradicts this assertion (184). Tacrolimus combined with IVIG or prednisone treatment greatly improved symptoms and reduced Ab titers in two patients who showed no response to other medicines (192). After 4 weeks of treatment with tacrolimus, serum anti-GAD Ab titers in patients with SPS were decreased, with an increase in motor ability, and the patients became completely self-dependent (191).

### 6.3. IVIG therapy

IVIG is the initial immunomodulator for patients with SPS with severe symptoms or unsatisfactory symptom improvements on other medications (193). IVIG therapy for patients with SPS partially improves symptoms (193) or the patient quality of life (194). It is also safe, with the duration of improvement being 6 weeks to 1 year (2).

### 6.4. Plasma exchange (plasmapheresis) therapy

Plasma exchange therapy is an option for patients with SPS who have failed to respond to other treatments (194). Plasmapheresis is usually conducted in one cycle with five sessions of plasma exchange. In a previous study, plasma exchange was used to treat two patients with SPS who had failed to respond to other treatments, resulting in improved symptoms and increased anti-GAD levels (195). Albahra et al. reported that among 10 patients with SPS, three had completely resolved symptoms, whereas seven had only partially relieved symptoms (196).

The outcomes of SPS treatment were reported to vary, resulting in a large range of improvements and moderate walking disability (21, 118). Limited reports have shown that patients with CA undergoing treatment have exhibited considerable improvements when assessed using the modified Rankin score. However, walking disability was still observed (197). Unfortunately, there were only modest outcomes for patients with LE following treatment (110, 138, 198–200), with symptoms, such as seizures and cognitive impairment, remaining (199).

### 6.5. Changes in autoantibody titers after treatment

#### 6.5.1. Anti-GAD65

After immune globulin therapy, 11 patients with SPS showed improvements in their movement disorder and decreased serum anti-GAD65 Ab titers (169). As we previously described, serum and CSF anti-GAD65 Ab titers were found to decrease, with those of CSF decreasing more rapidly than those of serum after patients with SPS received immunotherapy (138).

#### 6.5.2. Ovarian teratoma

A 26-year-old woman with anti-NMDAR encephalitis and SPS with an ovarian teratoma was successfully treated via laparoscopic removal of the ovarian tumor. She received immune-suppressant medications (methylprednisolone followed by a combination with baclofen) preoperatively and postoperatively, and her symptoms were gradually resolved (9).

#### 6.5.3. Breast cancer

A 53-year-old male patient had anti-amphiphysin-positive SPS and breast cancer, as previously mentioned. After undergoing surgery to excise the cancer, he received adjuvant chemotherapy with cyclophosphamide, methotrexate, and 5-fluorouracil, followed by post-mastectomy radiation and adjuvant endocrine therapy with tamoxifen. After 1 year of surgery, the stiffness in his upper extremity, but not his lower extremities, greatly improved (42). However, for a 30-year-old female patient with anti-amphiphysin, GAD Ab-negative SPS, and breast cancer symptoms were not alleviated following surgery (41).

#### 6.5.4. Lung cancer

A 56-year-old woman with anti-amphiphysin-positive SPS associated with small-cell lung cancer received treatment with benzodiazepines and corticosteroids, followed by cancer therapy with cisplatin/etoposide and radiotherapy. Following treatment, she exhibited signs of improved stiffness and was able to walk independently for short distances (72).

## 7. Animal models of SPSSD

There are some reports of SPS animal models (13, 113, 114, 201–209). For example, the animal models of anti-GAD65 SPS comprise two major types, specifically an *in vitro* animal tissue model and an *in vivo* animal model (13). Some studies have focused on *in vitro* (113, 114, 201–209) and *in vivo* SPS animal models (13, 132, 208, 210–213). Unfortunately, the results of these studies were not satisfactory, and further development is needed.

### 7.1. *In vitro* animal tissue studies

*In vitro* SPSSD studies are usually divided into three assays, enzymatic assays, whole-cell patch clamp recordings, and immunofluorescence-using cultures. The major samples in studies using enzymatic assays have been rat pancreatic islet extracts (201), crude rat cerebellar extracts (202), and recombinant human GAD65 (113). These studies demonstrated that high titers of GAD Abs are associated with SPS, whereas few cases (2/12) of high GAD Ab titers were reported in type I diabetes (202). Furthermore, the studies revealed that GAD65 can recognize conformational epitopes in the C-terminus (113).

The major samples for studies using whole-cell patch clamp recordings have been rat cerebellar slices (203, 204), rat hippocampal neurons (205), mouse hippocampal neurons (206), and rat hippocampal slices (207). These studies revealed that presynaptic GABAergic transmission is inhibited by GAD Abs in the CSF of patients with SPS and selectively suppressed (203, 204). In addition, these studies demonstrated that post-synaptic inhibitory potentials are increased by GAD-positive epileptic serum (205) but not by serum from patients with GAD65 Ab-associated LE (206) or with GAD65 Ab-associated epilepsy (206, 207). The major sample for studies using immunofluorescence based on cultures has been rat hippocampal neurons (114, 208, 209). These studies found that GAD Abs from some patients with SPS do not bind to the neuronal surface or that GAD Abs are not internalized by live neurons, suggesting the presence of other Abs specific to unknown antigens, rather than GAD (13).

### 7.2. *In vivo* animal model

The two major reported types of *in vivo* SPSSD animal models are passive transfer animal models, where transfer is induced using the serum or CSF antibodies from patients with SPS, and active immunized animal models induced using the human GAD65 protein (13).

#### 7.2.1. Passive transfer animal model

The main reported methods for passive transfer animal models using rats or mice are single cerebellar or paraspinal injections (132) and intrathecal (210, 211) or intraperitoneal injections (208, 211). Unfortunately, these animal models do not effectively mimic the clinical symptoms of SPS. However, some symptoms, such as paraspinal electrophysiological evidence of continuous motor activity (132), increased anxiety-like behavior (212), worsened rotarod results, and deficits in postural control (211), were partially matched.

#### 7.2.2. Animal model of active immunization

Active immunization using the human GAD65 protein has been effectively performed in type I diabetes studies; however, it has failed for neurologic diseases, including SPS, despite the high titers of GAD Abs (213) developing in these studies. This suggests that the GAD65 protein is also important for the pathogenesis of SPS; however, it is regulated by other autoantigens that contribute to the pathogenesis of SPS.

## 8. Conclusion

This review demonstrated that the relationship among cancers, autoantigens, and SPSSDs is complicated, and new information in this field is still being revealed globally. Our findings would facilitate the development of an open-minded approach to updating information on novel cancer subtypes, autoantigens, and SPSSDs to renew our database. Future investigations are urgently required to reveal the mechanism by which cancers, autoantigens, and SPSSDs interact, which will facilitate the early prediction of cancer outcomes and the discovery of new therapeutic modalities.

## Author contributions

YP received funding support and developed the research hypotheses. YP, HY, Y-hX, QC, HJ, SL, S-yY, and M-qD wrote the main manuscript. The final manuscript is the end product of the joint efforts of all authors. All authors contributed to the article and approved the submitted version.
